# Aldose reductase gene is associated with diabetic macroangiopathy in Japanese Type 2 diabetic patients

**DOI:** 10.1111/j.1464-5491.2006.01946.x

**Published:** 2006-08

**Authors:** A Watarai, E Nakashima, Y Hamada, G Watanabe, K Naruse, K Miwa, Y Kobayashi, H Kamiya, M Nakae, N Hamajima, Y Sekido, T Niwa, Y Oiso, J Nakamura

**Affiliations:** Department of Endocrinology and Diabetes, Nagoya University Graduate School of Medicine Nagoya, Japan; † Department of Preventive Medicine/Biostatistics and Medical Decision Making, Nagoya University Graduate School of Medicine Nagoya, Japan; ‡ Department of Clinical Preventive Medicine, Nagoya University Graduate School of Medicine Nagoya, Japan

**Keywords:** aldose reductase, atherosclerosis, macroangiopathy, polymorphism, stroke

## Abstract

**Aims:**

The aldose reductase (AR) gene, a rate-limiting enzyme of the polyol pathway, has been investigated as a candidate gene in determining susceptibility to diabetic microangiopathy. However, the association of the *AR* gene with diabetic macroangiopathy has not been investigated. Therefore, the present study was conducted to determine whether genetic variations of *AR* may determine susceptibility to diabetic macroangiopathy.

**Methods:**

There were 378 Type 2 diabetic patients enrolled in this study. A single nucleotide polymorphism in the promoter region (C-106T) was genotyped and the AR protein content of erythrocytes measured by ELISA.

**Results:**

There were no significant differences in genotypic or allelic distribution in patients with or without ischaemic heart diseases, but there was a significant increase in the frequency of the CT + TT genotype and T allele in patients with stroke (*P* = 0.019 and *P* = 0.012). The erythrocyte AR protein content was increased in patients with the CT and TT genotype compared with those with the CC genotype. After adjustment for age, duration of diabetes, body mass index, systolic blood pressure, HbA_1c_, and serum creatinine, triglycerides, and total cholesterol in multivariate logistic-regression models, the association between this *AR* genotype and stroke remained significant.

**Conclusions:**

Our results suggest that the CT or TT genotype of the *AR* gene might be a genetic marker of susceptibility to stroke in Type 2 diabetic patients. This observation might contribute to the development of strategies for the prevention of stroke in Type 2 diabetic patients.

## Introduction

There is familial clustering in the incidence of diabetes-related chronic complications [1], which suggests the existence of genetic susceptibility to these complications. Many studies have indicated that overactivity of the polyol pathway contributes to the development of diabetic microvascular complications by demonstrating the preventive effects of polyol pathway inhibition on these complications [2]. Aldose reductase (AR) is the rate-limiting enzyme of the polyol pathway, and thus polymorphisms in the *AR* gene may be one of the factors that determine genetic susceptibility to diabetic microvascular complications [3,4]. Recently, a new polymorphism, C-106T, at position −106 in the promoter region of AR, was identified and an association with diabetic microangiopathy in Caucasian and Asian subjects with Type 1 and Type 2 diabetes mellitus has been reported [5–12].

Other reports have indicated a role of polyol pathway hyperactivity in the development of diabetic macroangiopathy *in vivo*[13,14] and *in vitro*[3,15,16]. Thus, the *AR* gene could also be a predisposing factor to diabetic macroangiopathy. However, the association of the *AR* gene with macroangiopathy in diabetic subjects has never been reported.

To clarify these issues, the present study investigated whether the C-106T polymorphism of the *AR* gene determines susceptibility to diabetic macroangiopathy, such as ischaemic heart disease and cerebrovascular disease, in Japanese Type 2 diabetic patients and non-diabetic subjects.

## Patients and methods

We screened 417 consecutive patients attending Nagoya University Hospital. Patients with Type 1 diabetes (14), malignant diseases (13) and other types of diabetes (steroid or pancreatectomy induced; 10) were excluded. During screening, two patients declined to participate. A total of 378 Type 2 diabetic outpatients (28–88 years of age, 210 men and 168 women) were enrolled. A total of 334 non-diabetic subjects (17–89 years of age, 206 men and 128 women) who underwent a medical check-up in our hospital from April 2001 to December 2003 served as the control group. They had fasting blood glucose levels < 6.1 mmol/l and had no family history of diabetes. Five had an abnormal electrocardiogram and 20 a history of atherosclerotic diseases.

Assessment and definition of diabetic macroangiopathy was based on the following criteria. Cardiovascular disease was defined by a history of ischaemic cardiovascular diseases (e.g. previous myocardial infarction, angina, coronary-artery bypass grafting). Stroke (ischaemic cerebrovascular disease) was diagnosed by means of neurological signs and symptoms, together with computed tomography or magnetic resonance imaging. According to the Acute Stroke Treatment (TOAST) classification [17], only large-vessel diseases and carotid stroke were enrolled, and cardioembolic and lacunar stroke were excluded. Data regarding the presence of peripheral vascular disease (PVD) were also collected but the prevalence was too low to conduct statistical analyses, and is not included in this paper. The study protocol and informed consent procedure were approved by the Ethics Committee of Nagoya University Hospital and was performed in accordance with the Helsinki Declaration of 1975, as revised in 1983.

### Genotyping

DNA samples were prepared from whole blood using a QIAamp DNA Blood Mini Kit (Qiagen, Chatsworth, CA, USA). The C-106T polymorphism of the *AR* gene was determined by the polymerase chain reaction restriction fragment length polymorphism method using the primers and conditions described by Kao *et al*. [5]. The presence of the C allele was indicated by a 206-bp fragment and T allele was indicated by 147- and 59-bp fragments after overnight digestion with *Bfa-I* at 37°C. Before enzymatic digestion with *Bfa-I*, the product was purified with QIAquick PCR Purification Kit (Qiagen). The AR protein content in erythrocytes was measured by ELISA using anti-human AR monoclonal antibody (Mitsubishi Gas Chemical, Tokyo, Japan).

### Statistical analysis

Statistical analyses were performed using SPSS for Windows version 12.0 (SPSS, Chicago, IL, USA). Data are presented as means ± sd, unless otherwise indicated. Statistical significance of the differences between the groups was determined by chi-square (χ^2^) tests, unpaired Student's *t*-tests or anova where appropriate. The Hardy–Weinberg test was performed using a standard observed-expected χ^2^ test. Multiple logistic-regression analyses were used to assess the association of the CT + TT genotype with diabetic complications. Odds ratios (ORs) and their confidence intervals (CIs) were used to estimate relative risk. A two-sided *P*-value less than 0.05 was considered statistically significant.

## Results

The clinical characteristics of diabetic patients and non-diabetic subjects are presented in Table 1. The mean age, body mass index (BMI), fasting blood glucose levels, systolic blood pressure (SBP), diastolic blood pressure (DBP), and serum levels of triglycerides and creatinine of diabetic patients were higher than those of non-diabetic subjects. The high-density lipoprotein (HDL) cholesterol of diabetic patients was lower than that of non-diabetic subjects.

**Table 1:** Clinical characteristics of the study population

Variable	Type 2 diabetic patients	Non-diabetic subjects	*P*-value
*n* (male/female)	378 (210/168)	334 (206/128)	0.098
Duration of diabetes (years)	14.0 ± 9.5		
Age (years)	62.5 ± 11.9	48.8 ± 12.8	< 0.001
Diabetes treatment (D/O/I/I+O)*	58/203/72/43		
Body mass index (kg/m^2^)	23.4 ± 4.1	22.6 ± 3.1	0.002
HbA_1c_ (%)	7.4 ± 1.4		
Fasting blood glucose (mmol/l)	8.3 ± 2.7	4.9 ± 0.4	< 0.001
Systolic blood pressure (mmHg)	131 ± 16.8	122 ± 15.7	< 0.001
Diastolic blood pressure (mmHg)	74 ± 10.3	78 ± 11.3	< 0.001
Total cholesterol (mmol/l)	5.3 ± 0.94	5.2 ± 0.92	0.521
Triglycerides (mmol/l)	1.52 ± 0.90	1 ± 0.74	< 0.001
High-density lipoprotein cholesterol (mmol/l)	1.35 ± 0.40	1.46 ± 0.37	0.001
Low-density lipoprotein cholesterol (mmol/l)	3.2 ± 0.81	3.2 ± 0.85	0.809
Serum creatinine (mol/l)	82 ± 73.5	70 ± 14.1	0.004

Data are n or mean ± sd.

* D, diet only; O, oral glucose-lowering agents; I, insulin; I+O, insulin and oral glucose-lowering agents.

The genotype distributions of the C-106T polymorphism of *AR* gene were 259 (68.5%) for CC, 105 (27.8%) for CT, and 14 (3.7%) for TT in the diabetic group, and 213 (63.8%) for CC, 113 (33.8%) for CT, and 8 (2.4%) for TT in the non-diabetic group. The frequencies of the C allele were 82.6% in the diabetic group and 80.7% in the non-diabetic group. The distribution of genotypes was in Hardy–Weinberg equilibrium. There were no significant differences between the two groups (genotype χ^2^ = 3.708 with 2 d.f., *P* = 0.157; allele χ^2^ = 0.698 with 1 df, *P* = 0.404).

Because the number of diabetic patients with the TT genotype was only 3.7%, subjects with the CT and TT genotypes were combined for further analyses. The genotypes were not associated with gender, duration of diabetes, age, diabetes treatment, BMI, HbA_1c_ and fasting blood glucose levels, SBP, DBP, serum levels of total cholesterol, triglycerides, HDL cholesterol, low-density lipoprotein (LDL) cholesterol and creatinine.

Table 2 shows the distribution of the C-106T polymorphism in diabetic patients with or without ischaemic heart disease or stroke. Stroke was more frequent in patients with the CT and TT genotypes and T allele than those with the CC genotype and C allele. However, there were no significant differences in the prevalence of ischaemic heart disease between genotypes or alleles.

**Table 2:** Relationship between the AR genotype, allele and diabetic macroangiopathy in Japanese Type 2 diabetic patients

	Ischaemic heart disease		Stroke	
		
	–	+	–	+
Genotype				
CC	84.0 (204)	16.0 (39)	92.2 (225)	7.8 (19)
CT + TT	85.0 (96)	15.0 (17)	84.1 (95)	15.9 (19)
		*P* = 0.808		*P* = 0.019
Allele				
C	84.3 (493)	15.7 (92)	91.0 (534)	9.0 (53)
T	84.3 (107)	15.7 (20)	83.5 (106)	16.5 (21)
		*P* = 0.995		*P* = 0.012

Data are % (n).

*P*, two-sided χ^2^ test.

**Figure 1:**
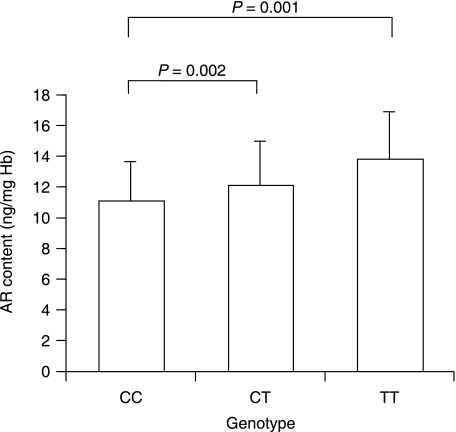
Erythrocyte AR contents of diabetic patients classified according to genotype of the C-106T polymorphism. Data are mean ± sd.

We then compared the AR protein content in erythrocytes among the genotypes. The AR content was significantly increased in a genotype-dependent manner (CC, 11.2 ± 2.5 ng/mg Hb; CT, 12.2 ± 2.8 ng/mg Hb; TT, 13.9 ± 2.9 ng/mg Hb, *P* = 0.002 and 0.001 vs. CC; Fig. 1). However, there was no relationship between AR content and presence of macroangiopathy.

Age (*P* = 0.003), SBP (*P* < 0.001), serum creatinine levels (*P* = 0.008) and serum levels of triglycerides (*P* = 0.048) were higher in patients with a history of stroke.

Table 3 shows the results of multivariate logistic-regression analyses with two models. After adjustment for age, SBP and serum levels of creatinine and triglycerides in model 1, individuals with the CT and TT genotypes were significantly more likely to have had a stroke than individuals with the CC genotype (OR 2.40, 95% CI 1.15–5.01, *P* = 0.019). Furthermore, in model 2, after additional adjustment for HbA_1c_, BMI, serum total-cholesterol levels and duration of diabetes, this relationship remained (OR 2.79, 95% CI 1.27–6.13, *P* = 0.010).

**Table 3:** Odds ratios for associations between risk factors and the prevalence of stroke with the use of multivariate logistic-regression models

Variable	Odds ratio (95% CI) Model 1	*P*-value	Odds ratio (95% CI) Model 2	*P*-value
Age (years)	1.06 (1.02–1.10)	0.002	1.06 (1.01–1.11)	0.009
Systolic blood pressure (mmHg)	1.03 (1.01–1.06)	0.011	1.03 (1.00–1.05)	0.053
Serum creatinine (µmol/l)	1.40 (1.03–1.90)	0.029	1.39 (1.01–1.90)	0.043
Triglycerides (mmol/l)	1.63 (1.13–2.36)	0.009	1.62 (1.07–2.45)	0.024
AR genotype	2.40 (1.15–5.01)	0.019	2.79 (1.27–6.13)	0.010
HbA_1c_ (%)			0.98 (0.70–1.37)	0.897
Body mass index (kg/m^2^)			1.01 (0.91–1.13)	0.802
Total cholesterol (mmol/l)			0.98 (0.63–1.53)	0.934
Duration of diabetes (years)			1.01 (0.97–1.06)	0.530

## Discussion

We have investigated the C-106T polymorphism in the promoter region of the *AR* gene as a candidate gene for susceptibility to diabetic macroangiopathy, and found that the CT or TT genotype is associated with increased risk of stroke in Type 2 diabetic patients.

Diabetes mellitus is one of the major risk factors of coronary heart disease and cerebrovascular disease. The high prevalence of these macrovascular diseases in diabetic patients can be explained by hyperglycaemia per se, as well as by the increased frequency of conventional risk factors such as hypertension, dyslipidaemia, and obesity.

Accelerated proliferation of vascular smooth muscle cells (SMCs) is one of the characteristic features of atherosclerosis [18], and it has been proposed that hyperglycaemia contributes to the hyperproliferation of SMCs [15,19]. Moreover, there are some reports that the inhibition of the polyol pathway by AR inhibitors prevents the enhanced proliferation of SMCs cultured in high-glucose conditions [15,16,20] and also prevents hyperglycaemia-induced NF-κB activation [21] which is involved in inflammatory signal transduction. We have previously shown that hyperactivity of the polyol pathway caused intimal thickening of coronary arteries in the galactose-fed dog, an animal model of polyol pathway hyperactivity, and that this abnormality was ameliorated by treatment with an aldose reductase inhibitor [13]. Taken together, these results indicate that polyol pathway hyperactivity may be involved in the hyperplasia of SMCs, and may play a substantial role in the development of diabetic macroangiopathy. However, previous studies have demonstrated that polyol pathway hyperactivity contributes to the development of diabetic microangiopathy, including neuropathy, nephropathy and retinopathy [2].

Hodgkinson *et al*. [22] reported that there is a significant correlation between increased AR expression, antioxidant gene mRNA levels in peripheral mononuclear cells cultured in high glucose and the CA repeat polymorphism of the *AR* gene in patients with nephropathy. Furthermore, the CT or TT genotypes and the T allele of this polymorphism have been identified as genetic markers of susceptibility to diabetic microangiopathy in several reports [5–12]. Therefore, genetic susceptibility to the development of not only microvascular but also macrovascular diseases in diabetes may be explained by the C-106T polymorphism of *AR* gene. However, a functional reporter gene assay reported by Yang *et al*. [23] showed that the promoter activity of a construct containing the C allele is higher than of a construct with the T allele in transfected HepG2 cells in high-glucose conditions. Further studies will be required to clarify these inconsistent results.

We showed that the CT and TT genotypes and the T allele of the C-106T polymorphism in the *AR* promoter region are thought to be a risk factor for stroke. Some independent effects of genetic variants (e.g. prothrombin gene, factor V gene, methylenetetrahydrofolate reductase gene, apolipoprotein gene) on the risk of cerebral ischaemia have been reported previously [24]. Compared with these gene variants, the adjusted OR we observed in this study suggests that the *AR* gene exerts a stronger effect on the risk of stroke.

Makiishi *et al*. reported, for the first time, the relationship between the C-106T polymorphism of the *AR* gene and erythrocyte AR content in Type 2 diabetic subjects [9]. The results obtained in this study that AR contents were increased in a genotype-dependent fashion (CC < CT < TT), are not completely consistent with their observation, but do support the hypothesis that polymorphisms of the *AR* gene influence erythrocyte AR content and the development of diabetic complications. Unfortunately, however, a significant relationship between the AR content in erythrocytes and the prevalence of stroke was not observed in our study.

Our findings should be cautiously interpreted because there are some limitations to the present study. Our analysis of the *AR* gene was limited to a single marker from the 5′ region of the gene. Thus, it cannot be excluded that this *AR* polymorphism is in linkage disequilibrium with an unidentified polymorphism strongly related to stroke risk. This could explain the lack of correlation between AR content in erythrocytes and the prevalence of stroke. Hyperglycaemia, hyperosmolality, and oxidative stress all regulate erthrocyte AR content [25–28], and also contribute to the development of diabetic complications including macroangiopathy. Therefore, the strength of the influence of these factors on erythrocyte AR content may explain our unexpected results

There are several well-known conventional risk factors for macrovascular diseases (for example, dyslipidaemia, hypertension, hyperglycaemia, and increasing age) but little is known about inherited factors that could predispose to diabetic macroangiopathy. Among the conventional risk factors, in the present study, age and systolic blood pressure were higher in the subjects with stroke than those without stroke, although the significance was weak after adjustment for various risk factors. In contrast, the CT + TT genotype and the T allele of the *AR* gene were associated with an increased risk of stroke, and the significance of these genotypes remained high even after adjustment for other risk factors. In addition, the erythrocyte AR content was higher in the subjects with these genotypes. Therefore, it may be speculated that polymorphisms of the *AR* gene, concomitant with an increase in erythrocyte AR content, would increase vulnerability of brain arteries resulting in stroke. However, prospective intervention studies with aldose reductase inhibitors would be required to confirm the influence of the *AR* gene polymorphism and AR content on the development of stroke.

In contrast, it is noteworthy that the influence of this polymorphism on the development of ischaemic heart disease is less than that on stroke. There are important differences in risk factors for stroke and ischaemic heart disease. For stroke, hypertension is the most adverse risk factor, whereas for ischaemic heart diseases LDL cholesterol is the strongest. This suggests that there are differences in the pathogenesis of ischaemic heart disease and stroke, which might explain our lack of association of the *AR* gene with ischaemic heart disease. It can be speculated that brain vessels are more sensitive to polyol pathway-induced hyperosmolar stress than coronary vessels. Precise investigation will be required to clarify this issue.

It is important to replicate our results in other populations. No previous study has investigated the relationship between diabetic macroangiopathy and *AR* polymorphisms in other populations. Although various studies have reported the relationship between diabetic microangiopathy and *AR* polymorphisms [5–12], consistent results have not been obtained, which may be as a result of differences between racial groups. Further study would be required to clarify this issue.

In conclusion, we have found that the C-106T polymorphism in the promoter region of the *AR* gene is associated with an increased risk of stroke in Type 2 diabetic patients. This observation might contribute to the development of further strategies for the prevention of stroke in Type 2 diabetic patients.

## Abbreviations

AR: aldose reductase
BMI: body-mass index
DBP: diastolic blood pressure
HDL: high-density lipoprotein
LDL: low-density lipoprotein
SBP: systolic blood pressure
SMCs: smooth muscle cells
